# Barriers to progressing through a methadone maintenance treatment programme: perspectives of the clients in the Mid-West of Ireland’s drug and alcohol services

**DOI:** 10.1186/s12913-018-3717-2

**Published:** 2018-11-29

**Authors:** Lisa Moran, Eamon Keenan, Khalifa Elmusharaf

**Affiliations:** 1 Graduate Entry Medical School, University of Limerick, Limerick, Ireland; 2 HSE, Social Inclusion Office, Primary Care Division, Dublin, Ireland

**Keywords:** Opioid use disorder, Opioid agonist treatment with methadone, Barriers to effective treatment, Clients’ perspective

## Abstract

**Background:**

The ‘perfect’ journey through an Irish Methadone Maintenance Treatment Programme (MMTP) would have a client engage appropriately with all relevant services available to them, inclusive of psychiatry, counselling, out-reach support, nursing and psychology. Concurrently, a client would ideally adhere to their prescribed methadone-dosing regimen, until a client is stabilised allowing them to function optimally. At this point, a client should transfer to the GP community setting. Unfortunately, this fails to occur. To date, very few studies have specifically investigated the reasons why a cohort of clients remain ‘trapped’ in the high risk, specialist clinical setting.

**Methods:**

Qualitative detailed semi-structured interviews were undertaken with 17 clients of one of Ireland’s Health Service Executive (HSE) Drug and Alcohol Services, entitled ‘*HSE Mid-West Limerick Drug and Alcohol Service*’. Each client had a severe Opioid Use Disorder (OUD) and clients had spent on average 7.5 years engaging with the MMTP.

**Results:**

Participants’ life journey prior to an OUD included Adverse Childhood Experiences (ACEs) and early exposure to illicit drug use. Shared life events resulting in their initiating and sustaining an OUD involved continuous hardship into adulthood, mental illness and concurrent benzodiazepine use disorder, often resulting in harrowing accounts of participants’ loneliness and lack of life purpose. Their living environments, an erroneous understanding of their illness and poor communication with allied health professionals further perpetuated their OUD. Positive factors influencing periods of abstinence were familial incentives and a belief in the efficacy of methadone. Clients own suggestions for improving their journeys included employing a multi-sectorial approach to managing OUD and educating themselves and others on opioid agonist treatments. If clients were not progressing appropriately, they themselves suggested enforcing a ‘time-limit’ on clients to engage with the programme or indeed for their treatment to be postponed.

**Conclusions:**

To optimise the functioning of the MMTP, three tasks need to be fulfilled: 1) Reduce the incidences of ACEs, 2) Diagnose and treat clients with a dual diagnosis 3) Educate clients, their families, the public and allied health care professionals on all aspects of OUD. A cross- departmental, inter-governmental approach is needed to address opioid misuse as a societal issue as a whole.

## Background

Opioid Use Disorder (OUD) is worldwide problem [1]. Globally, estimates indicate that 13.5 million people abuse or misuse opioids, including 9.2 million who have a specific dependence on heroin [2]. The universal encumbrance of OUD results from its associated health difficulties, incapacities and death [3]. Worldwide, in deaths implicating drug use disorders, opioids account for 76% [2]. In 2016, 10.6 million people worldwide were known to inject drugs and it is this cohort that endure the greatest health risks as greater than half of them live with hepatitis C, and greater than 10% live with HIV [4].

Most recent figures estimate that approximately 3 million people with an OUD reside in the United States [5]. European statistics reflect that there are approximately 1.3 million high-risk opioid users in the EU where opioids are found in 82% of fatal overdoses [6]. The most recent Irish data dates from 2006, and estimates that, at the time, there were approximately 20,790 opioid users in Ireland, a rate of 7.2 per 1000 [7].

It has become increasingly clear that changing the language of dependence is not just a matter of political correctness; terminology actually impacts clinical care [8, 9]. It has finally been acknowledged that to improve treatment and minimise the stigma surrounding OUD we must use first-person and medically precise language. However, if we truly wish to improve results, we should also amend the language of treatment [10]. Wakeman argues, and we agree, that the stigma surrounding the use of pharmacotherapy, in particular opioid agonist therapy, such as methadone, is more potent and harmful that the general stigma of addiction. The most widely held false belief is that opioid agonist medication is simply a ‘replacement’ or a ‘substitution’. To that end, we aim to use precise, respectful clinical terminology in this manuscript, including person-first language and to refer consistently to medication as a treatment, as in ‘opioid agonist treatment, as opposed to a ‘substitution’ or a ‘replacement’ therapy.

Heroin is the most consumed opioid contributing to OUD. It is a multi-faceted condition typically needing many different treatment modalities inclusive of pharmacological and psychosocial measures [11]. In Europe, 61% of clients receive methadone, resulting in it being the most frequently administered agonist therapy [6]. Methadone has ideal properties for the long-term treatment of OUD: Administered orally, it is absorbed slowly through the GI tract.

Typically, a single dose of methadone overpowers the symptoms of opioid withdrawal for 24–36 h. Methadone does not produce analgesia, sedation or euphoria [12]. It follows that the client can function in society without impairment and experience appropriate pain and emotional reactions. An additional benefit of methadone is that it over-ride cravings [13].

Over 50 years of research confirms that opioid agonist treatment with methadone (OATM) is a successful treatment for OUD [14]. Today, Cochrane reviews indicate strong evidence to support its use [15, 16]. The effectiveness of OATM in reducing HIV risk behaviour [17], Hepatitis C transmission [18], as well as overall mortality [19] is well established. In addition, the literature clearly shows OATM reduces crime rates [20], improves employment and family relationships [21] as well as quality of life [22].

Typically in Europe, specialist out-patient centres account for the single biggest provider of OATM in terms of client numbers. The second biggest source of OATM are health care centres. Included in this category are General Practitioners (GPs). In large countries such as Germany and France, these centres are central to the provision of treatment [6]. Ireland mirrors the current European structure where methadone has been prescribed since 1992. It is the most common opioid agonist treatment option. The 1998 legislation, the *Misuse of Drugs (Supervision of Prescription and Supply of Methadone) Regulations*, implemented a specific administrative structure designed to monitor treatment delivery and individual trends, the confidential Central Treatment List (CTL). The legislation also enforced a protocol for the prescribing of methadone, the Methadone Treatment Protocol, which provides for the delivery of methadone treatment in the Irish context. As in Europe, in Ireland, under this legislation, methadone is provided for in both specialist out-patient centres and in Primary Care Centres, both chiefly staffed by GPs.

Under the Irish MMTP, the ‘ideal’ journey through an Irish HSE Methadone Specialist Centre, such as the one in this study, would have a client engage appropriately with all relevant services available to them inclusive of counselling, out-reach support, nursing and psychology. Simultaneously, they should properly engage with their prescribing doctor and adhere to their methadone-dosing regimen, which is typically increased incrementally until a level is reached where clients’ OUD is stabilised to allow them function optimally in society. At this point, a client should be transferred to the care of a GP in the community who should provide the totality of their medical care including OATM. This would be beneficial from the perspective of both the client and the clinic. It would allow the client greater autonomy in their long-term treatment plan and allow the clinic to address the needs of those on the waiting list for treatment initiation. Unfortunately, many clients never appropriately stabilise to meet the above criteria.

In Ireland, at year-end 2016, there were 80 HSE Methadone Specialist Centres in operation treating 5438 clients [23]. Of those 5438 clients, only 117 were appropriately stabilized, and as such, transferred to the lower risk community setting [23]. This accounts for only 2.2% of the potential transferrable client population. Why do clients not stabilise and progress through the system appropriately? Is it behavioral? We must learn how clients view and understand their surroundings if their behaviour can ever be interpreted usefully. A benefit of qualitative studies is that it allows the researcher to analyse and grasp drug use from the clients’ perspective. To date, such studies have helped us to destigmatise drug taking and dispel negative stereotypes. Qualitative research also enhances our understanding of the theories of dependence and allows us to formulate and assess drug policy and practice [24]. Therefore, a qualitative analysis of the clients’ behaviours in this study should help us understand this failure in the system better.

Internationally, significant qualitative research with respect to clients’ perspectives of a MMTP has been published. Much research has been done focusing on ‘out-of-treatment’ individuals [25] and on retaining clients in treatment (a significant predictor of outcome) [26]. For those in treatment, qualitative studies focusing on clients’ access to treatment [27], level of influence on their treatment plan [28], quality of life [29] and their overall satisfaction in Methadone treatment [30] are well documented. However, very few of these studies focused on the long-term client failing to progress optimally through the system. Similarly, in Ireland, there has been a significant increase in qualitative studies attempting to address issues with OATM from the perspective of the client. Nationally, Ireland’s Drug Strategy (2009–2016) shone a light on the need for more service user participation and the creation of local and regional service user platforms. In an urban-based Irish study, Aoibhinn King, discovered that clients had little autonomy over the course of their treatment and had no representative role at service or task force level.

Her study suggests that despite the fact that providers understand the potential benefits of incorporating client experiences into the drafting delivery and assessment of services they remain ‘passive players’ in the equation [31]. Over the intervening time, Irish research has attempted to address this deficit. In 2012, Linda Latham reported on the experiences of service users receiving methadone treatment in urban general practice in Dublin and in so doing highlighted the positive influence of the GP setting in supporting recovery [32].

However, both internationally and nationally very few qualitative studies specific to the cohort, who remain in treatment but fail to progress optimally, have been conducted. To date, no Irish study has specifically investigated the reasons why a cohort of clients remains ‘trapped’ in the high risk, specialist clinical setting. These clients are likely the most complex but in studying their journey to and through the MMTP, we hope to establish patterns of similarity, which would flag their high-risk status on admission and allow us to intervene to optimise their care plan sooner. In doing so, we can optimise the efficiency of the service we provide. This study, ultimately, wishes to give the clients a voice in addressing this complex issue, as their experience of the service is ultimately, what will determine its success.

## Methods

### Methodological approach

The epistemological positioning underpinning this qualitative study is one of ‘social constructionism’; a term coined by Norman Blaikie who states; “*knowledge is neither discovered from an external reality nor produced by reason independent of such a reality. It is the outcome of people having to make sense of their encounters with the physical world and with other people*.” [33].

### Study participants and setting

At year- end 2016, 134 clients were receiving treatment with the opioid agonist, methadone in the Mid-West of Ireland’s specialist centre. Of these 134 clients, only 20 (15%) transferred to the lower risk, GP setting in the community [34].

The Methadone Specialist Centre of the HSE Mid-West Limerick Service operates 10 methadone clinics weekly. Each clinic monitors approximately 14 clients. Clients are randomly assigned to their individual clinic. The first author LM oversees three of these clinics, resulting in an average exposure to 40 clients weekly, with a male to female ratio of 3:1. Therefore, this client exposure reflects a good representative sample of the overall population of the clinic. The inclusion criteria for this study was that each participant had spent a minimum of 2 years in the specialist centre. As such, 24 of her clients were eligible for inclusion in the study.

Trust was integral to achieving an honest account of each participant’s lived experience. The first author LM worked closely and built a good rapport with all participants over the last two years. Therefore, the participants were selected purposively from LM’s client list, choosing those who had been longest on the programme first to participate in the study. Many of the potential participants were illiterate or had only very basic literacy skills so LM fully explained the information leaflet to them and asked them to bring it home where they could further review it with their families. After the information giving session, each potential participant was given an opportunity to think about his or her potential involvement and discuss the research with LM. A minimum period of one week was allowed before following up and asking them if they were willing to sign the consent form. All clients who were approached agreed to participate in the study.

### Sample size

The method of thematic analysis does not enforce conditions in relation to the size of a given sample. As such, three main factors shaped the sample size, the complexity of the data expressed by the interviewees, the appearance of shared themes during data analysis and the pragmatic restrictions of the main author, mainly time constraints. Accounting for the above, 17 of the 24 clients were interviewed of which 13 were male and 4 were female, reflecting a male to female ratio similar to the overall population of the clinic, 3:1. The 17 clients had spent on average 7.5 years engaging with the MMTP.

### Data collection

Semi-structured interviewing was the method employed to collect data for this study. This data collection method enables the researcher and the participant to engage in a real time discussion. It also provides a forum for original and unexpected issues to arise, allowing the researcher to subsequently investigate issues in more detail with further questions should the need arise.

#### Interview guide

In writing the interview questions, the authors were cognizant to safeguard participants’ ability to provide in-depth, complete accounts of their journey to and through the MMTP. Six key factors (see below) framed the broad structure of the guide but ensured it was malleable enough to permit the interviewer to follow-up and further explore interesting foci as they appeared. The interviewer met with participants at a time that was most convenient for them. A reminder phone call to each participant was made 24 h before the scheduled time.

#### Interviews

Individual comprehensive semi-structured interviews were conducted in person by the main author, LM, in a confidential office space situated in the Methadone Specialist Centre of the HSE Mid-West Limerick Service. At the outset, LM explained the voluntary nature of the interview to each participant. Their choice to withdraw at any point and an explanation of how confidentiality was going to be upheld throughout was also clarified before both participant and LM signed the consent form.

LM opened the interview with an icebreaker and then inquired as to the participants’: 1) Childhood & Education, 2) Early Adulthood & Criminality, 3) Drug History prior to heroin, initiation of heroin and current usage, 4) Current health, inclusive of mental health, 5) Current social circumstances (housing, employment, familial relationships), and 6) Engagement with MMTP (initial & current). The length of each interview varied, ranging from 10 to 47 min, with an average of 24 min across the 17 interviews. There were no follow-up interviews.

#### Audio recordings

Client names were purposely omitted from the recordings. The digital data was password protected before a professional transcribing company typed the recordings verbatim. An offer was extended to all participants to review their transcripts on their return but all declined this service.

##### Data analysis

Qualitative thematic analysis is a method for detecting, analyzing, unifying and recounting themes found within a data set [35] and as such is a perfect fit for this study. The inductive thematic analysis of this study, presented in Table 1 below, was undertaken using Braun and Clarke’s structure of six levels of analysis [35]. Firstly, data familiarization and code generalization was completed. Then theme search, review and naming was carried out. An inductive analysis of the themes was subsequently undertaken. The overall process itself was both iterative and reflective and involved a continuous ebb and flow between the phases. Finally, a summary report was generated.

**Table 1:** The inductive thematic analysis used in this study

Phase 1 Data Familiarisation	This involved the main author, LM, reading the first transcript closely a number of times and checking the transcript back against the original audio recording for accuracy. Each review of the recordings provided some new understandings and LM began taking personal notes focusing on content, language use, context, and initial interpretative comments.
Phase 2 Initial Code Generalisation	LM coded interesting features of the data using the computer software NVivo for qualitative data administration. LM worked methodically through the full data set. She gave complete and uniform consideration to each item, tagging and naming selections of text with each data item.
Phase 3 Theme search	Merging and deviation of themes within the data were noted, leading to the development of the next phase, transforming codes into emergent themes. This involved LM working more with her notes rather than with the transcript and again inputting findings into the NVivo software package.
Phase 4 Theme review	LM firstly ensured all coded data extracts formed a coherent pattern and then progressed to considering the validity of each individual theme
Phase 5 Theme naming	LM scanned for links between emergent themes, assembling them according to conceptual similarities. Each cluster was then allocated a descriptive label. Using NVivo allowed for short descriptions of themes and subthemes, using links to appropriate passages in the transcript.
Phase 6 Final Report	LM produced a report of the analysis undertaken.

Subsequent to this initial assessment, a further sixteen interviews were undertaken. A similar analysis of the data from these interviews was conducted. Themes were reconfigured and re-labelled. Analysis of the 16th and 17th interviews revealed no new data and as a result, no additional interviews were deemed necessary.

##### Ethical considerations

Participating clients are dependent on their prescribing doctor to receive their weekly methadone prescription and as such, there is an obvious unequal relationship between them and the first author, LM, which warrants further explanation. To address this understandable bias, prior to conducting the research, the main author spent 2 years deliberately building a rapport with these clients, ensuring they trusted her and were fully aware of her ethical motivations in conducting this research, which ultimately aimed to improve the delivery of the MMTP to better address their needs. In doing so, LM ensured to the best of her ability that client participation was both optional and truthful.

The potential risks were minimal. However, it was acknowledged that there was a risk of emotional distress for the participant during the interview given that they will be talking about the challenges that are inherent in being a long-term client of a MMTP. The main author and the Multi-Disciplinary Team MDT worked together to make sure to minimise this risk and dealt with any upset that occurred both immediately and in the longer-term by offering regular follow-up counselling services. Reassurance of confidentiality were given and participants were assured that they could cease the interviews at any point if they so wished. As their clinician, the first author having the overall responsibility for their health from a Methadone perspective monitored the health of participants during the study.

Following the University of Limerick Records Management and Retention Policy, E-transcripts were stored on a password -protected computer and hard copies were locked in a cabinet in the main author’s office. University Hospital Limerick’s Research Ethics Committee on December 13th 2016 granted full ethical approval for this study: REC Reference 131/16.

## Results

On initial analysis, an enriching insight into the personal journeys of each client to and through the Mid-West of Ireland’s MMTP emerged from the data. Common themes across their life journey prior to an OUD were documented. Shared life events resulting in their initiating and sustaining an OUD were also captured. Subsequent analysis revealed both negative factors perpetuating their continued opioid usage and positive factors encouragingly influencing periods of abstinence. Both sets of factors were recorded. Finally, clients own suggestions for improving their journeys were identified.

### Personal journey to and through the MMTP

**Fig. 1:**
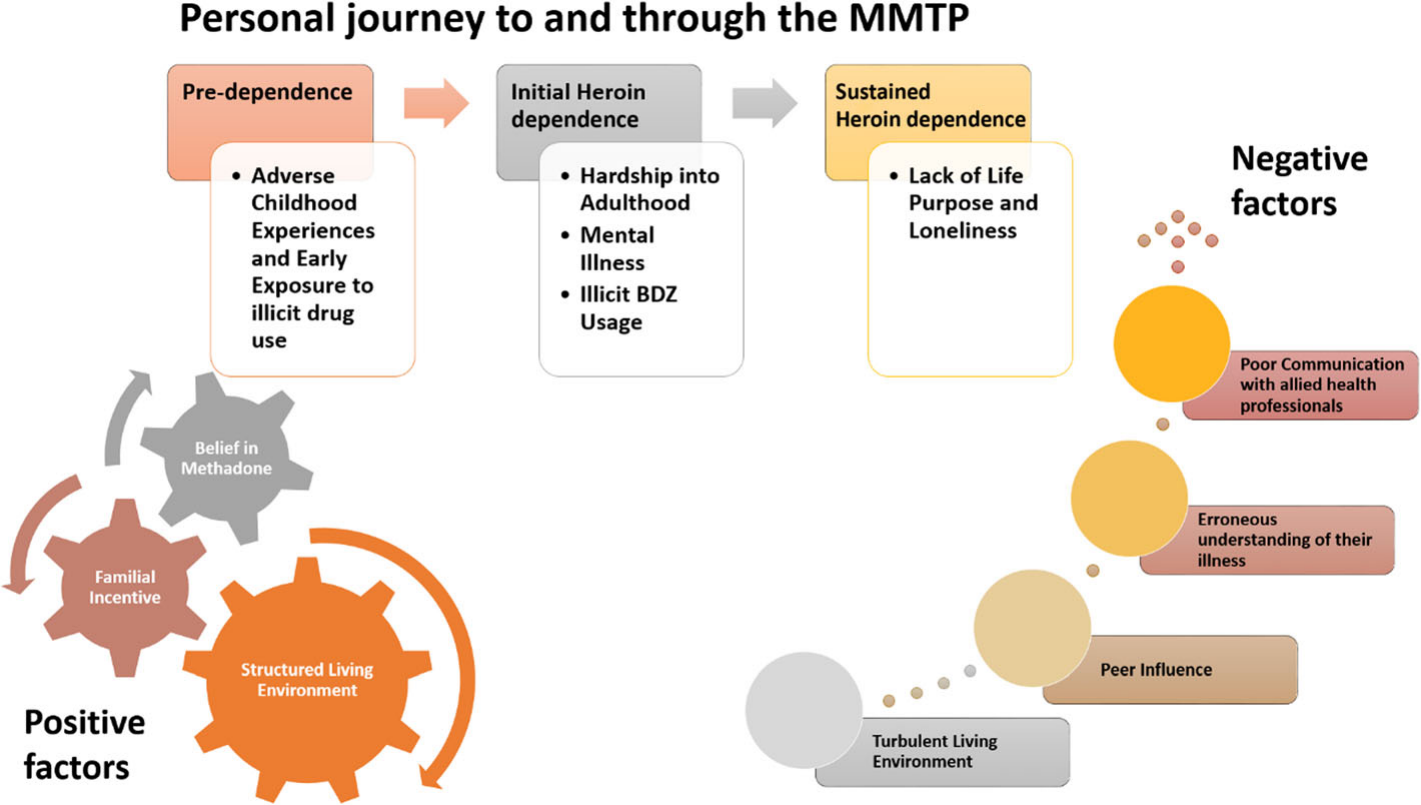
Clients’ personal journeys to and through the MMTP in the HSE Mid-West Limerick Service

Their journeys, though each unique, had common chronological sub-themes, which are schematically shown in Fig. 1 and elaborated on below.

#### Adverse Childhood Experiences & Early exposure to illicit drug use

Childhood adversity was an exceptionally common occurrence across the interviewees, occurring both inside and outside the home. Reports of both physical and verbal abuse were common, with particular emphasis on paternal physical abuse. Alcoholism was also reported to be prevalent within the home, which was often coupled with reports of domestic violence. The clients documented further reports of experimenting with cannabis, gas, butane, LSD and solvent abuse. Mental illness permeated these households adding to the adversities of childhood. As a result, some clients were placed in the care of the State.

*“I was in care for a bit & stuff. My mam died and my father couldn’t handle me. I had psychoses*.” Interviewee 14

Abuse, outside the home was also common, both for those in institutional care

*“Life there was horrible, horrible, horrible, horrible, some terrible things, Oh I don’t even want to talk about them...”* Interviewee 9

and for those living in the general community

*“When I was young like there used to be a man with a black fluffy dog the whole time. I would always see him like, but he was just a bad man. Then he did bad things to me”.* Interviewee 17

Reports of bullying outside the home and parental separation within the home were also documented. Having a family member in incarceration was also common. Coupled with this, most interviewees reported exposure to a culture of illicit drug use from a very young age.

*“When I was 10 or so, my mother and my father were working. I used to go into the drinks cabinet with an empty bottle of lemonade and fill it up with all different spirits…. When I was about 12 or 13, me and my friend found a load of Roche 30s [flurazepam monohydrochloride tablets of 30mg strength] …. we took the full box between us. And then I ended up I woke up in St. Johns hospital… they said that like my heart stopped for a couple of seconds.” Interviewee 12*

#### Continuous hardship from childhood into adulthood

Misfortune and difficulties continued to permeate their lives from childhood onwards. Often, their inability to cope with these relentless stressors resulted in commencement of heroin abuse and indeed frequently perpetuated its continued use thereafter. The brief excerpts below summarises the struggles clients face on a daily basis and explain their regular relapses with addiction.

*“My sister got murdered, she got shot dead. The hit was meant for me. My other brother got stabbed to death…. My other brother, he done 9 years in prison and he got out and died in the shower of a heart attack…. My little brother just died, meningitis in the brain got him. My other brother he is a schizophrenic. He’s in and out of prison. He lives in a mental hospital now*. *The most stable influence in my life was the presence of my father. Unfortunately, he passed away in 2016, when I was in incarceration” Interviewee 11*

#### Mental illness, inclusive of psychoses, depression and anxiety

More frequently than not, reports of mental illness were recorded in tandem with these clients’ heroin addiction. Psychoses, depression and anxiety disorders were the most common mental illnesses documented. These illnesses may have been present from a young age, as interviewee 2 reports, *“I suffered from psychosis since the age of fifteen. Schizophrenia psychosis I have*” or indeed brought on later in adulthood as a result of harrowing adversities.

*“I lost two children in 9 days. My son, he hung himself in prison, three days left in his sentence. My daughter committed suicide 9 days later. She went out, bought Xanax (alprazolam), and took the whole lot and she took about nine bags of heroin as well and just threw herself off the bridge. Afterwards, I tried to kill myself.” Interviewee 9*

Clients often expertly captured their misdiagnosis by health care professionals due to suffering from both their heroin dependenceand a mental illness. They might feel that their psychiatric team only focused on their dependenceand not depression and mental illness.

*“Yeah, I had depression there when I was out on bail for killing, that really got me down. I was in the acute psychiatric unit for about two, three months. They [the psychiatric team] were, they were mixing the two for me, they were trying to say that it was all got to do with the drugs and everything in my own heart and soul like it was because of what happened, you know”. Interviewee 3*

#### Illicit benzodiazepines usage

Not altogether surprisingly, an overwhelming proportion of interviewees were dually addicted to benzodiazepines (BDZ). More often than not clients were buying these on the city’s black market. Due to the high demand for BDZ outstripping local supply, the market was becoming more dependent on foreign suppliers, and purchasing them on the internet was commonplace. Clients have no idea what they are purchasing, as these tablets are illegally manufactured and so do not resemble prescribed BDZ in shape or size and indeed are often laced with other drugs.

*“At the moment, Benzos are a big thing in Limerick. They’re Spanish supposed to be, now there’s two different types, there’s ones that say U94 on them. You can bust them in four, they’re supposed to be two milligrams and the shape of them is very funny, I’d say it would be very hard to copy them though. And then there’s other ones that says Xanax on the front of them, but they’re real thick and chunky and they have two on the back, they’re supposed to be two milligrams as well, but I took them and they made my urine dirty here”* [A ‘dirty’ urine refers to a urine-testing positive for opiates] *Interviewee 7*

The use of BDZ to enhance the effect of heroin was also apparent. Chaotic, alarming use of BDZ was evident, where the risk of accidental overdose was undisputed.

*“I put them [BDZs] into the pot and inject them with the heroin so I inject them both together” Interviewee 12*

*“I am after taking 6-7 sticks* [Sticks are a potent form of street Xanax, approximate equivalent to 2 mg Xanax] now [before interview]. *I could take 20-30 of them a day. I buy sticks over Xanax because they are stronger. They are sent back from Spain; they are charging a fortune for them. I take Upjohn 90s* [1mg Xanax] *as well, maybe 10-15 a day” Interviewee 15*

Often, their supply of BDZ was from the same supplier as their heroin and as such, BDZ usage perpetuating heroin usage was evident. The cost of feeding their dual dependencealso surfaced as a stressor in their lives.

#### Lack of life purpose and loneliness

Clients expressed a significant lack of purpose in their lives. Boredom was a dominant reference. and was described as a reason for their continued heroin use.

*“Boredom is a big part I think, just sitting down at home with nothing to do thinking I will smoke that now it might knock me out” Interviewee 16*

Loneliness was a prevailing emotion linked to this subtheme. Lack of employment, a known contributor to the economic burden of this disease, had a significant negative impact on clients’ self-worth. Many blamed having a criminal record for their unemployment. Inability to work due to disabilities related to their heroin abuse was also evident. Lack of motivation was also evident.

*“It’s very hard, motivation is lacking big time, yeah. I’m on disability at the moment, it’s because I’ve had DVTs in both legs over injecting [into the groin]. I don’t think I am fit enough to work… and with a criminal record” Interviewee 3*

### Negative factors which influence clients’ journeys

#### Turbulent living environment

On analyzing the data, three specific unstable living conditions emerged which perpetuated the continued use of heroin, living with an addict, living in hostel accommodation and finally being homeless. Living with an addict resulted in clients stating they often sought heroin on behalf of their partners as opposed to fueling their own habit. This was reported as a ‘safety’ measure as they worried if their partners attempted to seek heroin on their own behalf they would end up in trouble, be it, assaulted themselves by mixing with an undesirable crowd or committing a crime, such as robbery to fuel their habit on their own.

*“I would be hoping that it don’t come through like but if I don’t get it he would probably end up robbing something, do you know I mean? So it’s actually protecting him by getting it off my sister. Keeping him in doors and away from danger” Interviewee 1*

Homelessness was also reported as an environment that fueled their need to continuously use. Motivation was hugely lacking in the homeless cohort, as being homeless secludes you from many basic needs.

*“If you haven’t got your own address you can’t get any help, medically, doctors, on assistance on anything. No, you can’t even get the dole. That is one of the main reasons I continue to use” Interviewee 2*

Finally, the environment most associated with continued use or frequent relapse was for clients living in hostel accommodation.

*“I’m in a hostel now and there’s two people dealing fucking heroin. It’s full of addicts. I can’t open my eyes, I can’t go outside my room without bumping in to someone and they’re either talking about drugs or doing drugs or a way of getting them, and it was just a matter of time before my brain... gives in” Interviewee 3*

#### Peer influence

Data analysis confirmed that clients cited the term ‘friend’, ‘mate’ or ‘cellmate’ as the method by which all were first introduced to heroin. Though all lives differed greatly on their individual journeys, peer influence on initiation of their addiction, was the one constant variable. Thereafter, continued friendships with other drug takers perpetuated their dependence.

*“I don’t really have any friends who don’t take drugs” Interviewee 16*

The only potential solution cited to address this issue was to attempt to completely isolate themselves from their social scene.

*“I’ve isolated myself from all other addicts like, do you know, I don’t talk to any of them, I don’t, I’ve blocked all the drug dealers’ numbers”. Interviewee 6*

However, given that the majority of these clients live in hostel, shared accommodation with other drug addicts isolating themselves from their peers is near impossible as is evident from the quotes in the ‘turbulent living conditions’ section above.

#### Erroneous understanding of their illness

Uniformly, clients had a poor understanding of the chronicity of their illness, the likelihood for long-term OATM or indeed of the rationale for using methadone as a medical intervention in their disease management. Clients were asked to reflect on how long they thought they would need treatment for when they first engaged with the programme. Their answers varied from 3 to 6 months. However, in reality, the average time spent in OATM across the 17 clients was 7.5 years. That said, when asked re the likelihood for them needing ‘long-term’ opioid agonist therapy, the vast majority of clients were resolutely confident that they would not require ‘long-term’ medication. Over the last 12 years, interviewee 4, had repetitively engaged and re-engaged with the programme, relapsing regularly during the time but when probed as to why he believed he would not need ‘long-term’ therapy answered confidently, *“I think I’m passed that, you know, I think I am”.*

Clients’ confidence in remaining opiate free was disproportionate to their actual achievement. At the time of interview, interviewee 11, had succeeded in not using heroin for just one week but was adamant she would remain opiate free forever more. Not only so, but because of her week’s sobriety she now wished to cease methadone completely. When probed if she knew of anyone who had successfully ceased methadone and remained opiate free thereafter she denied knowledge of it. Yet was confident in her own success without methadone.

*“Well I am clean now a full week. It’s my first time I am clean and I am staying clean…. I don’t want it* (the methadone) *I am sick of it, it’s not for me… To be honest with you. I can’t think of anyone there that came off Methadone and stayed clean” Interviewee 11*

When asked re their level of education with regard to methadone itself participants were uniformly poorly educated. Education levels of clients’ families were also poor, which in turn resulted in lack of familial support for the treatment.

*“I got given a leaflet, so I just read through that, you know.’ (I learned) a little bit off YouTube” interviewee 8. “No, no one explained to me how methadone works” Interviewee 9*

*“My mam, she hates the stuff, she hates it because she thinks it’s the Devil’s drink” Interviewee 7*

Interviewee 12 was embarrassed by the fact that the public was, *“paying taxes for keeping people on methadone”.* When challenged and asked if the public should not view opiate dependence as a chronic illness, much like diabetes, and requiring methadone was much like diabetics needing insulin he defended the public’s perception of heroin dependence:

*“You are not born with the choice of diabetes but you are born with the choice of not taking heroin” Interviewee 12*

#### Poor communication with allied health professionals

The majority of clients reported a fragmented, poor and sometimes fractious relationship with their General Practitioners. They reported the fear of being stigmatised as the reason for hiding their heroin dependenceand engagement with the MMTP from their GP.

*“I didn’t tell my GP I had a heroin problem because I, I wasn’t telling anybody that I had a heroin problem. I was so anxious and nervous that I was going to get judged” Interviewee 8*

However, on finding out the client was on the MMTP their relationship immediately disintegrated. The GP recalled all the times she prescribed opiate based medications for the client because of his reported pains and began to doubt the need or truth in his needing it. As a result, she felt she could no longer remain his GP. As a result, the client reported feeling abandoned and isolated from the health care system.

*“I felt like a complete, excuse me, piece of shit. When I walked outside that door I never felt so lonely and so scared in my life’.... I’ve no GP from then till now” Interviewee 8*

Reports of a similarly tenuous, disjointed relationship with local counselling and mental health services were documented. Furthermore, poor inter-communication between allied health professionals was also evident. Interviewee 12 reported suffering with panic attacks. His GP referred him to the local psychiatric hospital where benzodiazepines were prescribed as a treatment modality. However, he reports his GP not agreeing with this treatment plan.

*“When I came back to my GP he then took me off the benzos. I told him I still get panic attacks but he said, “it’s all in your mind” and to just exercise your way out of it like” Interviewee 12*

### Positive factors, which influence a clients’ journey

#### Structured living environment

Often, the more regularised their living conditions were, the greater their likelihood was of remaining opiate free. Three specific environments were tabulated; prison, hospital and when housed in a drug free environment. Interviewee 13 reported finding the structure and governance that prison offered, *“behind a steel door”*, a welcomed reprieve from his chaotic life which fueled his drug habit.

Reports of purposefully getting caught committing crimes to ensure imprisonment were documented, as clients knew the strict regime of prison had the potential to help them succeed in their quest for sobriety.

*“ I was using gaol as a rehab, do you know, I was going out, committing crimes and getting caught on purpose just to go in to gaol to get off the gear. I couldn’t get off it you know on my own outside. Gaol saved me enough of times. It worked for me. It gave me structure that you don’t have on the outside” Interviewee 4*

Interviewee 11 felt prison had saved her life. She was also so desperate to escape the clutches of her dependencethat she voluntarily presented herself for incarceration.

*“If I didn’t go to prison, I would have been found dead to be honest with you. I handed myself into the prison or else I would have fell to the ground and just died” Interviewee 11*

In addition, when given the opportunity to move from hostel accommodation, as documented above, to a drug-free apartment successful cessation was noted.

*“Ever since we got in to that apartment, we had no one around us that was on heroin, so I think that’s what kind of helped us” interviewee 5*

Other ‘controlled’ environments where successful reprieve from their dependenceoccurred was when hospitalised for prolonged periods or indeed when housed in a specific drug-detoxification centre.

#### Familial incentive

At various points in their journeys, these clients had periods of sobriety driven by certain incentives, mainly family members, particularly children or younger siblings. Their dependence results in their journeys being chaotic and quite often unmanageable but their overall ultimate wish in life is for simplicity. Their aspirations are basic, a normal familial home with full access to their children, where the can function as a stable parent and ultimately gain the respect of their children, siblings and other family members.

*“My ultimate goal is to, just to have my family back around me and get the house back to normal” interviewee 4*

Interviewee 12, was motivated by his daughter’s acknowledgement that her classmates were referring to him derogatively as a *“junkie”.*

*“That was kind of like one of the nails in the coffin for me, it was like going, Jesus Christ I had better get off this stuff…. But it’s an uphill struggle” interviewee 12*

Interviewee 11 did manage to remain opiate free when in prison and is aware of the benefits of a structured routine, as a result feels a detoxification centre may be her best option to rid herself of her addiction.

*“I would go into treatment at some point. To get my kids back, to live normally, just being a normal mother for my kids, that’s all I want” interviewee 11*

#### Belief in methadone

An extremely favorable relationship with regard to the pharmacological properties of methadone was reported, particularly in the early phases of treatment. The reports ranged from the very basic to the more complex. Clients were exceptionally grateful for methadone’s ability to prevent the symptoms of withdrawal, often giving a very honest description of the positive effects with regard to this. Beyond this basic function, other clients viewed methadone as a step closer to a more ‘normal life’ as it releases them from the grip of their disease and allows them to choose their path in life. Some viewed methadone as quite simply, lifesaving.

*“It just gives you time, it gives you a choice, methadone actually gives you the choice to take or leave heroin” interviewee 3*

*“I went on methadone because if I didn’t I would’ve ended up dead”. Interviewee 7*

### Clients’ personal proposals for improving their journey

Final data analysis identified clients’ personal proposals for improving their journey. At the end of each interview, each client was given the opportunity to suggest changes they would implement to the programme to optimise its effectiveness. The most common client derived suggestion was contentious as it involved limiting client time on the programme.

#### Enforce time limits on phases of their journey within the MMTP

Interviewees suggested a very strict induction phase of treatment for new clients with a well thought out plan to enforce it. All new clients would be limited on the programme to a 5-week treatment plan, increasing their dose incrementally by 5mls to a maximum of 50mls.

*“If you don’t bring back a clean sample after this, that is it for you, we will talk to you next month. We have someone else in line who needs to try this. Try it that way and see if it works or if it doesn’t” Interviewee 2*

Other clients not only supported the above measures but took it one step further by suggesting to also increase the weekly urine testing to twice weekly admitting that once weekly testing can be orchestrated by the clients to give false negative samples. When questioned as to the effect such sanctions would have on their own engagement with the programme most clients felt such sanctions would be inappropriate for their particular journey, citing *‘long-term’* engagement in the programme as a justifiable reason not to adhere to same.

*“But when you get to fifteen to twenty years like I am using methadone you just need it” interviewee 14*

#### Employ a multi-sectorial approach

Clients strongly advocated for the integration of their opioid agonist treatment with other relevant services, such as psychiatric services, GP medical care and housing services. The primary advantage to a unified approach is that clients attending the clinic would have easier access to these services thereby improving engagement with these supports.

*“I just think ye yourselves doctors, and psychiatrists, and housing* [representatives] *if you could roll all that into this clinic it would be fucking dynamite. Can you image the amount of people in Ireland you would be helping? The organisation you create would be phenomenal it would be phenomenal to be supported by the government” Interviewee 2*

#### Provide education

This subtheme imbued many of the transcripts. References were made to educating the public and a specific suggestion was to revise our wording of methadone as a ‘substitute’ as this terminology had exceptionally negative connotations and clients believed as a result, fueled the publics’ misunderstanding of the treatment.

*“Educate people, because when people hear you’re on methadone they just assume you’re on heroin and they don’t see it as a treatment, they see it as a substitute” Interviewee 8*

Clients also felt society’s youth needed to be properly educated re the dangers of drug misuse.

*“Oh Jesus the kids definitely need to be educated. A lot of these kids wound up taking drugs they didn’t know’* [what they were taking]”. *Interviewee 12*

## Discussion

The aim of this study was to gain insight into how the current treatment of OUD could be improved for those who fail to progress appropriately over time from the acute clinical setting to that of the local community GP setting within the Irish MMTP. As is evident above, data analysis highlighted many themes contributing to this failure but unfortunately, to sufficiently address all of these themes is beyond the scope of this manuscript. Therefore, for the purpose of this research manuscript, we will focus on further exploring three specific themes; ACEs and stress in later adulthood, dually diagnosed clients and education. The reason for focusing on these themes is that they focus on one aspect of pre OUD, childhood adversity and its impact on coping with stress in later adulthood, one during OUD, concurrent mental health illness and finally one which addresses not only the individual but the need for their families, the general public and health care professionals to better educate themselves on all aspects of OUD if the burden of this disease is ever to be addressed appropriately. After expanding on each theme specific recommendations as to how to ameliorate them are then suggested.

### Adverse childhood experiences (ACEs) and stress in later adulthood

The CDC study was the original and remains one of the most comprehensive research study on childhood neglect and abuse and their impact on subsequent health and well-being in adulthood [36]. It showed a definite causal relationship between the depth of exposure to family dysfunction or abuse during childhood and many of the risk factors for the leading causes of adult mortality, inclusive of illicit drug abuse. Nearly all clients in our study identified with childhood trauma.

Over the intervening 20 years, ACEs continued to be of increasing international concern and consequently there is a continuously growing wealth of research, which validates that chronic stressful experience in childhood, can lead individuals on a health harming life course, inclusive of illicit drug use [37]. Studies specific to illicit drug use continue to find remarkably high percentages of childhood trauma, specifically emotional, sexual and physical abuse, in drug dependent clients. The risk of early experimentation with substance abuse increased 2–4 times for each ACE and nearly 2/3rds of injection drug use can be linked to ACEs [38].

1n 2015, Public Health Wales distributed an internationally validated questionnaire to 2028 Welsh adults. It examined their current health behaviours and their exposure to ACEs [39] using an internationally validated questionnaire [40]. Compared with no ACEs, those with 4 or more ACEs were 16 times more likely to have experimented with crack cocaine or heroin. As is evident from our study above, many clients experienced adversities in childhood and all nine of the adversities listed in the Welsh study were documented in the transcripts in this study inclusive of, Sexual abuse, Physical abuse, Verbal abuse, Domestic Violence, Parental separation, Mental illness, Alcohol abuse, Drug abuse and Incarceration.

Children of those affected by ACEs are at a heightened risk of exposing their own children to ACEs [41]. This perpetuation of ACEs is commonly known as the ‘cycle of violence’. [42]. This continuous cycle can lock generation into OUD. It follows that stopping ACEs in one generation or minimising their impact on children can help not only those individuals but also their offspring. Such a cycle of childhood adversity can lock successive generations of families into opioid dependence. Consequently, preventing ACEs in a single generation or reducing their impact on children can benefit not only those individuals but also future generations. Research shows that there is a significant reduction in opioid use amongst adolescent patients receiving OAT which further compounds the need to intervene as early as possible in their treatment [43]. Public Health will play a central role in breaking the cycle of violence but they will require help and support from health care services such as Drug and Alcohol Services [39].

Trauma in early childhood impacts how we respond to stress throughout our lives and as stress plays an integral role in developing and sustaining dependence [44], it merits a brief look here. Exposure to early stressors in life, such as, poor parenting, family dysfunction, and adverse neighbourhood characteristics creates a lower “set point” for a child’s internal stress system.

It has been found that early life trauma can alter the brain’s stress regulatory system, which influences an individual’s ability to regulate emotion and respond to fear [45]. Consequently, individuals may be more vulnerable to health harming behaviours in later adulthood. This predisposition is developed further when trauma is subsequently encountered in later life [46].

If an individual has a heightened stress response, they are likely to attribute a high worth to substances that offer temporary relief such as opioid misuse. In contrast, activities, which typically offer satisfaction, such as, meaningful, familial relationships, are undervalued because in the client’s life they have never been fulfilling [47]. It is well established that as dopamine levels decrease, the craving for drug use increases. Stress reduces the function of dopaminergic receptors in the emotional circuits of the forebrain [48] and consequently increases the long term craving for opioids. The reward value in drug use is enhanced by stress and even after periods of abstinence, stress can provoke relapse [49].

In the treatment of OUD, incorporating a focus on stress and its management could improve treatment outcomes [44]. There is a considerable public health cost associated with drug use and such improvements would pay a considerable dividend.

### Dual diagnosis

Dual diagnosis is, as defined by the Royal College of Psychiatrists, 2002*, “the co-existence of both mental health and a substance use disorder including both drugs and alcohol”* [50]. Each disorder in itself is chronic and relapsing, travels an independent course and is capable of influencing the other disorder. Individuals experimenting with recreational drug use are more at risk for developing an OUD if they have a separate psychiatric condition [51]. Likewise, compared to the general population, clients diagnosed with OUD, have a greater risk of developing an independent psychiatric disorder, including a dependence on other substances [52]. As was evident in the analysis of the transcripts from our study, there is a complex relationship between the two as diagnoses range from 1) A primary psychiatric disorder with secondary substance use disorder, 2) A primary substance use disorder with psychiatric complications, 3) A concurrent substance use and psychiatric disorder, and 4) An underlying traumatic experience resulting in both substance use disorder and mood disorder.

Implications of a dual diagnosis are far reaching. Co-occurring psychiatric and SUDs are notoriously difficult to manage clinically. Adherence to treatment and its subsequent effectiveness are negatively impacted due to the comorbidity and ultimately service user morbidity and mortality are increased [53]. For clients with a dual diagnosis, optimizing treatment of their psychiatric disorders is essential to improving the outcome of their opioid use disorder. Benzodiazepine (BDZ) usage was particularly chaotic in the clients of this study. Research indicates that a comorbid tranquilizer use disorder is linked to higher rates of persistent opioid use [54]. Screening and addressing a co-morbid BDZ use disorder, in collaboration with clients’ GPs and psychiatric team members should improve their clinical outcome. Furthermore, the earlier in life one begins to use opioids, the increased likelihood of presenting with a concurrent psychiatric illness [55]. Awareness of this risk posed should better our ability to identify clients at an increased risk of dual-diagnosis in our clinical practices.

Two main barriers in addressing the needs of dually diagnosed clients lie in firstly diagnosing the problem and secondly collaborating with relevant stakeholders to ensure their treatment plan is optimised. A chaotic lifestyle is a contributory factor in failing to diagnose co-existing needs. This can affect individuals making and adhering to medical appointments or even availing of community-based services. The result of which is that those whose opioid use coexists with a mental health often reach crisis point. The existing health and social care system needs to change. Services are ill equipped and under resourced to deal with more than one problem at a time. Instead, the system responds to support clients’ primary need be it drug, alcohol or mental health. Many services are provided by the NGO sector who may be working in isolation from statutory providers.

### Education

The neurobiology of dependence is exceptionally complex and as a result poorly understood, even amongst professionals in the medical community. Clinicians need to impart evidence-based truths with regard to the addictive process in opioid use disorder to not only their clients but also their clients’ families and the public to optimise the functioning and overall success of any MMTP. Developments in our understanding of the neurobiological processes that arise following chronic and acute opioid administration have helped enhance our scientific understanding of how dependence develops.

We must focus on the individual and not his or her disease in the treatment of OUD. However, grasping an understanding of the neurobiology of drug dependence can be extremely valuable to the clinician. It can offer an insight into individual behaviours and problems, help establish person centred attainable goals and define the rationale for treatment [56]. Individuals who are taught about the origins of dependence can benefit from understanding the biological basis of their illness and its need for long-term, often life-long therapy to negate its effects centrally. The term ‘psychoeducation’ in opioid dependence refers to a form of communication between clinician and client that acknowledges the client’s role in understanding and dealing with the realities of their illness. The overall purpose to get the client to willing adhere to their treatment regimen while reducing or counteracting the factors that contribute to relapse.

Clients in treatment are typically driven by acutely pressing conditions such as the symptoms of withdrawal and have limited insight. They understand that craving is the leading contributory factor for relapse but lack long-term disease awareness, which means they deny the chronic nature of their dependence. Clients live in the here and now and solely focus on their immediate circumstances. They equate spontaneous short-term abstinence with full remission and any subsequent relapse is viewed as an isolated episode [57].

As is evident in our study it is when clients are relieved of their acute discomfort is when insight into their dependence regresses. They believe they are able to manage their cravings and opioid use. They struggle to accept any relapse prevention perspectives and fail to see drug dependence as a chronic relapsing disorder. The main aim of psycho education is to develop a higher level of client insight and understanding.

It was apparent in the above study that clients had many ‘misconceptions’ about OATM. Given our understanding of the neurobiology of drug dependence and the effectiveness of methadone in its treatment clinicians should actively engage in psycho-education to prevent relapse.

Despite the overwhelming evidence that OUD is an enduring, relapsing brain disorder the stigma associated with opioid agonist treatment, such as methadone, is clearly prevalent in today’s society. Unfounded opinions about ‘addiction’ are widely rooted in the cultural mainstream and are particularly harmful as they confound clients’ misconception of their illness.

## Conclusion

There is no denying the toll of OUD is tremendous. In giving a voice to the most complex of clients within the Irish Drug and Alcohol service, this manuscript highlighted many aspects of their lives before and during treatment, which need to be addressed in order to optimise their quality of life. In doing so, this may well reduce the burden of OUD on society as a whole, for the individual, their families and our communities. This manuscript explored three specific areas of redress, ACEs, dually diagnosed clients and societal education.

The MMTP is ideally placed to work collaboratively with public health, to access the most vulnerable and high risk of individuals subjected to ACEs and offer additional supports to meet their complex needs. To address stress in later adulthood, we recommend resourcing specific treatments for the management of stress in clients with an OUD, which will have far-reaching benefits. If we are ever going to effectively treat OUD, tailored interventions for the treatment of clients’ stress from psychologists, counsellors, psychiatrists and General Practitioners (GPs), all working collaboratively, are needed.

Goals for effective change for dually diagnosed clients should focus on collaboration between and education of all stakeholders (both statutory and non-statutory). It is vital that mental health staff receive drug and alcohol awareness training. Similarly, staff of the drug and alcohol services should receive mental health training. This would enable staff to better refer and work collaboratively. Information sessions offered by counsellors and psychologists would be beneficial. Multi-disciplinary forums would allow staff to tease out challenges and capitalise on opportunities with clients. The advantages of well-trained staff are obvious as inevitably this will improve results and make for a more efficient health care system.

From an education perspective, we must ensure to educate clients appropriately as to the neurobiology of their illness, the pharmacokinetics of methadone and its role in OUD. We must as Dana Hunt argued over 20 years ago, *“change the view of methadone maintenance within the heroin using community from that of a passive process of “giving up” to an assertive lifestyle of active recovery”* [58].

Another strategy that could be adopted is to create and disseminate short information packets for use by outreach services to counter ‘street myths’ on methadone and other OATs in dependence. Ensure that scientifically grounded information on methadone, as a treatment modality for OUD is included within the in-service training programmes of all clinics. Integrate information on OUD treatment into the curricula of national medical schools. Provide regular training and update sessions for not only General practitioners in the community but all allied health professional training programmes involved with the drug and alcohol services on the treatment of OUD and its advancements.

Public awareness as to disease process of dependence as well as the therapeutic benefit of treatments such as methadone must be publicised. Create, roll out and assess a public education campaign incorporating the voice and face of the ordinary person on opioid dependence and its treatment. Increase access to information on OUD and its treatment in mainstream health care facilities [59].

A cross- departmental, inter-governmental approach to address substance misuse as a societal issue as a whole is needed. Subsequent work needs to be done on addressing vulnerable children’s exposure to illicit drug use, concurrent BZDs use in individuals with OUD, their housing conditions and their lack of life purpose and loneliness.

## Abbreviations

MMTP: Methadone Maintenance Treatment Programme
OATM: Opioid Agonist treatment with Methadone
OUD: Opioid Use Disorder
